# Challenges newly-arrived migrant women in Montreal face when needing maternity care: Health care professionals’ perspectives

**DOI:** 10.1186/s12992-016-0229-x

**Published:** 2017-01-25

**Authors:** Sandra Peláez, Kristin N. Hendricks, Lisa A. Merry, Anita J. Gagnon

**Affiliations:** 1 Department of Educational and Counselling Psychology, Faculty of Education, McGill University, Montreal, Canada; 2 McGill Global Health Programs, McGill University, Montreal, Canada; 3 School of Nursing, University of Ottawa, Ottawa, Canada; 4 Ingram School of Nursing, McGill University, Montreal, Canada

**Keywords:** Migrant, Case study, Canada, Health services, Healthcare providers, Pregnancy, Childbirth

## Abstract

**Background:**

People who leave their country of origin, or the country of habitual residence, to establish themselves permanently in another country are usually referred to as migrants. Over half of all births in Montreal, Canada are to migrant women. To understand healthcare professionals’ attitudes towards migrants that could influence their delivery of care, our objective was to explore their perspectives of challenges newly-arrived migrant women from non-Western countries face when needing maternity care.

**Method:**

In this qualitative multiple case study, we conducted face-to-face interviews with 63 health care professionals from four teaching hospitals in Montreal, known for providing maternity care to a high volume of migrant women. Interviews were transcribed and thematically analysed.

**Results:**

Physicians, nurses, social workers, and therapists participated; 90% were female; and 17% were themselves migrants from non-Western countries. According to participants, newly-arrived migrant women face challenges at two levels: (a) direct care (e.g., understanding Canadian health care professionals’ expectations, communicating effectively with health care professionals), and (b) organizational (e.g., access to appropriate health care). Challenges women face are strongly influenced by the migrant woman’s background as well as social position (e.g., general education, health literacy, socio-cultural integration) and by how health care professionals balance women’s needs with perceived requirement to adhere to standard procedures and regulations.

**Conclusions:**

Health care professionals across institutions agreed that maternity care-related challenges faced by newly-arrived migrant women often are complex in that they are simultaneously driven by conflicting values: those based on migrant women’s sociocultural backgrounds versus those related to the implementation of Canadian guidelines for maternity care in which consideration of migrant women’s particular needs are not priority.

Sganarelle. As-tu bien la hardiesse de t’opposer au médecin? Hors de là!

Lucas. Je me moque de ça.

Sganarelle. Je te donnerai la fièvre.

Molière (1999). *Le malade imaginaire*. Hachette, Paris.

## Background

International migration is defined as a “movement of persons who leave their country of origin, or the country of habitual residence, to establish themselves either permanently or temporarily in another country” ([1], p. 52). There are approximately 232 million international migrants worldwide [2]. Half of all births in Montreal [3] and over one-fifth of births in Canada [4] are to women who migrated to Canada from other countries. This trend is projected to continue, and it has been estimated that by 2031, 47% of Canadians will have at least one parent born outside Canada and belonging to a minority group [5]. Despite the constant growth of migrant communities in Western countries, research depicting the relationship between migrant women and maternity care is scant. We therefore explored health care professionals’ perspectives of challenges newly-arrived migrant women to Canada coming from non-Western countries face when needing maternity care in order to better understand clinical practices towards these women.

### Health care inequities

Migrant women’s access to health care requires attention, especially during the first years of migration when adaptation to the receiving country is taking place [6]. Despite an international call to recognise both the health of migrants as a human right and the need for migrant-sensitive health policies and programs [6, 7], due to health care inequities, some migrant women arriving in high income level countries may: (a) face multiple health risks; (b) not receive the health care they need; and (c) face, along with their infants, multiple health care-related risks [8].

Health care inequities, observed in health care differences in the services provided to and received by two or more groups that are translated to health outcomes, represent a complex phenomenon [9, 10]. Factors related to health care inequities have been classified into three main levels pertaining to: (a) the patient, (b) the provider, and (c) the health care system. At the patient level, the most common factors include: source country language different than that of the receiving country; conditions associated to the movement itself; legal status; length of time in the receiving country; financial and administrative hurdles; lack of transportation to health care centres; reports of stigma, discrimination, and social exclusion; socio-cultural barriers; isolation; fear of harming chances for successful resettlement; previous negative experience with the health care system; and refusal of treatment or decision of leaving health problems unaddressed [11–18]. Also, at the provider level, clinical practices embedded with racism and ethnic biases have been consistently reported from health care professionals towards migrant women [13, 19–21]. Finally, at the health care system level, the most notable factor associated with health care inequities was the existence of regulatory restrictions on access to state-finaced health care [22].

While presenting the factors associated with health care inequities by levels is informative, it creates the impression that the majority of factors hindering the appropriate care of migrant women pertain to the patient level [23]. Instead, building on reports analysing governmental responses to populations in vulnerable conditions, we could argue that health care inequity of migrants relies on the complex interaction of different factors determined by the existence of socio-culturally non-inclusive norms and lack of policies in the receiving country [22, 24].

### Theoretical framework

Post-colonial theories argue that during colonial times, European ethno-centric reference norms related to individual and collective social experiences such as gender, education, religion, and race were established and supported. Values not conforming these standards were questioned and various forms of inequity and oppression were fostered [25]. These norms cannot be analysed as purely separate entities because they are seen as mutually reinforcing and interacting to preserve traditional colonial values [26, 27].

In the field of health, post-colonial theories have been used to identify and understand health care inequities, as well as to advance health care professionals’ knowledge. These theories have shown that different minority groups have been marginalised by institutions that hold colonial values. It has been argued that these groups should be repositioned and empowered within a culturally safe environment that listens to their voices; considers their knowledge; respects and recognises their values, beliefs and rights; and makes institutions more inclusive [26, 28–31].

Newly-arrived migrant women coming from non-Western countries represent a minority group whose values and standards have been often questioned [8]. Some health care professionals, guided by the perceptions they have of their patients, may use the power associated with their position to revindicate certain values that contribute to health care inequities [27, 32–34], creating a distance between “us” and “them” (i.e., their patients) [35].

## Methods

### Research design and epistemological stance

We designed a qualitative multiple case study [36] with the goal of gathering rich and detailed information about health care professionals’ perspectives of the challenges newly-arrived migrant women face when needing maternity care. We examined (within-case analysis) and compared (cross-case analysis) four maternity care units, focusing on the ‘how’ and ‘why’ of contextual factors that influence the health care professionals’ perspectives and assumptions concerning the referred population. We used a post-colonial conceptual framework referred to in the introduction to guide our study.

### Participants and procedures

After obtaining ethics approval from the Research Institute of the McGill University Health Centre, we purposefully selected four Montreal teaching hospitals to be our ‘cases’ based on the high volume of births to international migrants attended in their maternity units [3]. We aimed to interview ten key informants from each hospital working in maternity units (antepartum, birthing centre, and postpartum) having different professional backgrounds and work roles.

A nurse manager at each institution helped us identifying potential interviewees who were contacted via telephone, e-mail, and/or in-person. Those who agreed to participate were asked to suggest other informants whose viewpoint or experience would represent an important contribution to the study. Therefore, we combined purposive and snowball sampling to ensure a varied range of informants were included [37].

### Data collection

The first author conducted face-to-face, semi-structured, open-ended interviews [38] to discuss challenges, as perceived by health care professionals, newly migrant women experience when needing maternity care. As we were committed to learn the logic underlying health care professionals’ perspectives, we respected the interview schedule to compare data across the four cases, but we adopted a flexible attitude towards exploring other ideas raised by interviewees [39].

Interviews were conducted in the participants’ language of choice (English or French) and lasted approximately one hour (*M* = 58 min; range = 25–150 min). A second follow-up interview with the nurse manager at each hospital was conducted 4–8 weeks after the first. The purpose of this interview was to present preliminary data, share preliminary results, elicit additional information, and discuss whether more interviews were needed.

Eighty-seven percent of participants accepted to have their interviews audio-recorded. During those interviews, observational notes were taken. Immediately following the interviews, the interviewer listened to the recordings and wrote memos reflecting the thoughts and potential venues of analysis. These memos were then compared with the observational notes. Interviews were transcribed verbatim. Transcripts were verfied against tapes for accuracy, corrected if needed, and stored in a secured place. In cases in which participants did not agree to be recorded, we took copious notes during the interview that were used for analysis.

### Data analysis

Guided by our research question, we followed the analytical procedures proposed by Ritchie and Spencer [40]. This analytic approach relies on five interconnected stages: (a) familiarisation, (b) identification of a thematic framework, (c) indexing, (d) charting, and (e) mapping and interpretation.

As data collection was conducted in parallel with data analysis, we identified key concepts and explored them in-depth in subsequent interviews; thus, *familiarisation* with the material consisted of reading the transcripts to have both a sense of the material collected and to adjust, if needed, the interview guide. Then, we proceeded to the identification of a preliminary framework reflecting participants’ perspectives as well as theoretical discussions on the topic [22–24, 26, 30, 32–34, 41]. We used this analytic structure for *indexing the data*, a procedure most commonly referred to as coding (e.g., [42]). By doing this, we identified certain patterns and initial associations among them. As while indexing we were looking to answer contextual (i.e., identify the form and nature of what is already in place) and diagnostic (i.e., examining the reasons for, or causes of, what exists) questions, we searched for both single codes and narrative units (i.e., stories that allowed us to reconstruct participants’ perspectives). We then proceeded to the *charting* step, which entailed organising data previously synthesised and abstracted into themes by lifting it from its original context and arranging it depending on established relationships. Following this step, we compared the data within each case (i.e., participant by participant) and between cases (i.e., each case compared to the others). This practice allowed us to track and contextualise issues related to power, status, and professional roles that emerged across participants’ narratives and across cases [43]. Finally, we proceeded to the *mapping and interpretation* step that consisted of redefining concepts by comparing and contrasting key dimensions, themes, and patterns; creating typologies by linking dimensions and themes; and finding associations among themes.

Throughout the analysis we wrote analytical memos that were used to develop our ideas, organise our reflections, and support the presentation of evidence. A qualitative software package, MAXQDA (VERBI GmbH Software, version 10, Germany), was used to code and organize the data.

As a research team, we engaged in collaborative analysis by sharing a constant dialogue in which we discussed our positions, viewpoints, biases, and interpretations [44]. We also created a vignette to illustrate participants’ voices and presented it to different colleagues who gave us feedback and contributed to reshaping it. This triangulation of perspectives, mainly done via the discussion and review of both field memos and analytical memos, enhanced our self-reflection during the analysis [45]. The analytic meetings were subject to an audit trail that we used to support our evolution towards a more abstract and conceptual representation of data.

## Results

### Participants’ characteristics

Although initially we aimed to interview ten participants per institution, we continued the interviews until it was agreed that data were sufficient. Sixty-three participants in total were interviewed (Case 1, *n* = 13; Case 2, *n* = 15; Case 3, *n* = 15; Case 4, *n* = 20). Ninety percent of participants were female and 17% were themselves migrants from non-Western countries. Participants’ professional backgrounds and roles included: three family doctors, five obstetricians, four medical residents, one nutritionist, one anesthesiologist, seven social workers, one art therapist, one psychologist, one spiritual consultant, and 39 nurses.

### Health care professionals’ perceptions of challenges migrant women face regarding maternity care

We identified three main themes across participants’ discussions. Under the first theme, *women’s start-up conditions at the time of needing maternity care*, we grouped issues that referred to how the integration in the receiving society was mediated by participants’ characteristics, such as how women’s Canadian status represented an obstacle or facilitated access to the health care system. According to the participants, the length of time migrant women have been living in Canada was a key component that smoothed the challenges these women faced due to adaptation to the demands and characteristics of the receiving society. The second theme dealt with participants’ perspectives concerning *challenges migrant women face concerning health care*. Examples included balancing personal expectations and socio-cultural beliefs; establishing an effective communication with health care professionals; and having support within the health care system (e.g., a person helping with translation) to better follow the consultations. Also, challenges related to dealing with the heath care system were included in this theme. Examples of challenges related to the health care system included understanding how to: (a) navigate the system, (b) get access to standard care, and (c) effectively receive necessary care. The third theme, *Canadian standards of maternity care*, referred to the principles, procedures, actions, measures, and standards that are driven by Canadian health care guidelines, national and provincial laws, and case-specific institutional policies, missions, and responsibilities guiding health care professionals’ clinical practices. According to the participants, these health care standards, while aimed at providing the best response to the patients’ medical condition, do not necessarily respond to the individual’s psychosocial needs.

Keeping in mind that existing health care inequities affecting minorities is a complex phenomenon [46], we portrayed the findings including both the participants’ and the researchers’ voices [47] using a narrative writing approach, and more specifically, a nonfiction first-person, figurative, vignette [48]. The purpose of the vignette was to develop a compelling, insightful, and meaningful account [49, 50] illustrating data as voiced by participants and perceived by researchers [51]. As we began to understand the health care professionals’ perspectives through the interviews, the meaning behind their reasoning and behaviors -sometimes hidden behind the complexity of the phenomenon and embedded in the participants’ discourse, [39] became clearer for us. The vignette compiled participants’ perspectives resulting from both lifted raw data (i.e., in vivo words, phrases, features of participants’ speech [e.g., moments of silence]) as well as rephrased participants’ contributions resulting from our interpretation and representation of recreated meanings. As “[m]aking choices is a part of the composing process” ([52], p. 22), we decided to put together most significant participants’ contributions we identified when charting data, juxtaposing participants’ voices and our voices, and articulating our theoretical interpretation and explanation of the phenomenon.

### Health care professionals’ perspectives regarding challenges newly-arrived migrant women in Montreal face when needing maternity care: a vignette of study findings

*So you want to discuss ‘what are, according to me, the challenges newly-arrived - migrant women go through at the time they need maternity care,’ ok? Well, that’s a tricky question and I don’t have a single answer because, first, we are not talking about a homogeneous group as there is a tremendous heterogeneity among them. And second, I don’t know if it is pertinent to discuss this here because it goes a little bit further from your question, but I would say that in my experience, newly-arrived migrant women, even if they are close to delivery, have many things in their minds other than giving birth, such as housing, food, clothing, and education for their children. Often, migrant women tend to secure their families and kids first. They become the pillars of their family in terms of organisation, which is terribly difficult to accomplish because you usually have no money and, on top of that, you are pregnant, you have other kids to look after, or you’re looking for a job, or you have to go back to university because your degree isn’t valid here. So just that is a burden itself and there are associated factors that may facilitate or complicate your job, such as how well you master the language, how well-educated you are, or even, how familiar you are with the implicit, tacit, and invisible social rules.*

*Now, thinking aloud while trying to organise my ideas, I would say that the differences you see among these women turn around two main threads: The reasons for coming to Canada, including the country from where you’re coming; and the length of time these women have been living here. So basically, it’s not the same when you come to Canada from the US or France and you planned it, sometimes even as a family option, than if you’re coming as a refugee from a non-Western country. In the first case, you are prepared to face certain challenges that you may have even anticipated, because you’ve gone through similar things in your own country, such as a job search; whereas in the second case you are escaping from an abusive situation, you are probably poor, coming from a violent country, and leaving behind traumatic stories of suffering, repression. In addition, the language spoken in the receiving country has no similar roots, not even the same alphabet; the culture is completely different and as you didn’t anticipate coming here, you’re not necessarily prepared to face it; winter is challenging… So the truth is that in both cases you come looking for better options, thinking that you’re finally in a free and well-organised country, but you are not equipped to face the challenges. This said, the time living in Canada plays a crucial role because if you’ve been living here for a while, sooner or later you get more acclimatised to the culture and you end up by getting into the system and your family’s basic needs are already attended by then. We see a big difference, you know, between the ones that are just arriving and the ones that are already established.*

*And again, socio-cultural integration, which is a major issue, depends on the circumstances that brought you here. If you come from any country outside Canada, another thing that has to be taken into account is the Canadian status with which you are entering the country. So if you are, for example, a ‘perm’[anent]-resident, you may struggle to settle down, but as you will navigate the legal side of the system, your stuff will be moving quickly; however, if you don’t have a Canadian status, well, you will have no rights at all, they have literally nothing, not even access to legal recourses because they cannot even claim for refugee status. These people, it’s sad what I am going to say, but they just live in the shadows, in all possible senses!*

*And for migrant women who are pregnant, the ‘Canadian status’ is crucial because that opens or closes access to free health care. If you have a ‘Canadian status,’ no matter which one, you get access to care. Now, if you don’t have access to health care, no matter what, you can come and deliver, there is no question about it. But they have to pay, you know? So we don’t deal with them anymore, just because the hospital now asks for money upfront for deliveries. But we used to have undocumented immigrants, a while ago, not now. Those people are still around, but we don’t see them because they have to pay for everything, even for prenatal screenings. But Oh! Gosh! They’re around. You know what? I think the ‘package,’ they call it ‘package’ or something, I think it could be something like $10,000 to deliver here. If there is a chance of a caesarean section, if they have to have an induction which lasts so many days, if they are in pain and need an epidural, that’s extra I do believe. Well, this is what I am saying, there are a lot of people that come here with ‘no status’ and that means they have to pay the hospital and usually they don’t have that money. But they come here and they deliver. We get caught there because the ‘admin’[istration] says: “We will charge these people” and so they go out. What happens after I don’t know; Where do they go after? No idea, but they don’t have the money to pay and if they want to deliver, they have to pay upfront. But that is the case, as it is everywhere around the world.*

*And there are other challenges, besides access to the health care system. Probably the most salient ones being the sociocultural beliefs, the language, and the availability of social support. These three factors are crucial, and are difficult to deal with, from both sides I would even say; I mean, also for us. Maybe it’s me, but I think that the sociocultural beliefs are the toughest ones because you may need proper language to ask for the appointments and you may count or not on some family or friends to explain to you how to get into the health care system, unknown to you, that’s true. But now, when it comes to sociocultural issues it’s really hard for them because in addition to not having the language and feeling isolated, the only way you have to be emotionally linked to your family is by means of your rituals, your traditions, your beliefs. And for different reasons, usually genuine reasons, we have a hard time to respect them. The more typical ones are the need of the placenta to be planted, burnt, or eaten depending on different rituals; and the request of having just female staff around them. And we cannot please them with these requests because of the law and regulations: First, here in Quebec, all sample materials extracted are saved here at the hospital for safety reasons, so you may like it or not, we cannot give you the placenta; second, because of gender equity and because this is a teaching hospital, you will be appropriately served, but by whoever is available. And you sign for that when you come to deliver.*

*And the story does not end there… No… Once you understand how the system works and how to get into the system, there is another whole package that is getting the same health care Canadian women get. I think we are pretty spoiled here in Montreal, because we live in a multicultural city, but it may not be the case in other parts of Quebec. So for example, prejudices from health care professionals? Well, that’s another issue… Black people are always late, Latinos are not able to be responsible, and you keep on going… So picture this: a woman comes here, speaks no English or French, has her other two kids around her because she’s not yet working so they don’t go to daycare and her husband is working… So she’s not a priority because if she gets the care, who is going to take care of the kids? Just practical issues, you know.… I frequently ask myself whether these prejudices are going to stop… We tried education; we trained some of our professionals on multicultural issues… Well, it minimised the negative impact, but it is still there. If there is a clue coming out from this study, I would like to know what is it, if there is anything else we can do, that would be helpful to know.*

*So there’s a huge clash between these women’s culture and our health system. We follow evidence and when you don’t have the language or the experience, you probably won’t understand that we are doing it for your benefit. I know… You will say research evidence is mainly rooted in Western countries, that’s true, I give that to you, but that’s so far what we have as a credible account. So if I put myself in her shoes, and yes, they might feel we are pushing them sometimes.*

*Is there anything else I can say? I don’t think so, I am pretty sure I covered the whole spectrum. It’s interesting because even myself I always think of these women as a final outcome, as women coming for health care, and as a health care professional, I say to myself: “Just be fair and provide equal care.” Now, you pushed me to think about this… What is equal care? Is it the same we give to everyone, but does not respect these women’s stories? Or is it a different type of care we are talking about? If this is it, truly, more than being guided by my intuition I really don’t know what to do because there are so many factors conflating that I really don’t know how to proceed.*

*Funny thing, you know, you came with your questions and now I’ve got some questions of my own!*

## Discussion

In discussions held with health care professionals, three main themes were identified and related to: (a) women’s start up conditions at the time of getting pregnant and/or delivering the baby, (b) challenges migrant women face regarding maternity care, and (c) the relationship between Canadian standards of maternity care and women’s experiences regarding pregnancy and delivery. According to participants interviewed in this study, maternity health care challenges newly-arrived migrant women go through are varied and cannot be considered in a vacuum; instead, these challenges are part of a complex reality these women have to face. The navigation of the health care system is itself challenging because of the complex interaction of factors related to the patient, the health care professionals, and the health care system, as proposed in previous literature [22, 23, 53, 54]. In addition, these women are challenged by starting a new life in a new country (e.g., mastering the official language spoken in the receiving country) that, depending on their personal and social experiences, as well as contextual circumstances (e.g., availability of interpreters), may be (or not) addressed. Most of the challenges these women face are due to social and health care inequities and these women’s wellbeing is tightly associated to responding to these challenges from different perspectives (i.e., individual and health care system level) [55].

Comparisons showed no differences neither across the four identified cases, nor between participants who were themselves migrants from non-Western countries and participants who were non-migrants. Participants expressed their empathy towards migrant women’s efforts to fit into the Canadian standards of maternity health care, but at the same time they questioned women who refused to adopt or follow proposed standards of care, due to sociocultural beliefs. The participants agreed that it was key for migrant women to be acclimatised to the Canadian culture and to understand the health care system requirements [25, 27]. Of interest, the participants did not refer to women’s demographic characteristics as health determinants, but instead to how acculturation, assimilation, or integration [56], described as closely related to the length of stay in the new country, were crucial to these women to navigate both the social system and the health care system, a finding already reported [57]. In line with previous research, it seems that the health care professionals perceive the challenges migrant women go through, but at the same time, justify or reinforce the rules imposed by the health care system [58].

Another trend observed across given responses, and represented in the vignette, was that when participants showed more empathy and understanding towards women’s circumstances they referred to them as “you,” in a familiar way, while talking to the interviewer, whereas when they were pointing out aspects women should work on to fit Canadian standards of health care, they were referring to these women as “they,” creating a distance. Building from previous evidence, this seems to suggest that the standard norms underlying the medical culture are deeply ingrained in the health care system so that health care professionals assume medical standards as being unquestionable and perceive as outsider as those who are not aligned with these norms [26, 35].

As illustrated in the vignette, the description the participants used to present the events was not linear, and instead, unfolded across many different situations [59]. Participants fluctuated between perceiving migrant women as being in different circumstances and requiring different approaches, to perceiving them as local people who might be familiar with the health care system and therefore be deserving of equal and standard health care [60]. Thus, even when health care professionals may not necessarily contribute to health care inequities, they may be reinforcing values deeply embedded and promoted in professional socialisation and training mainly based on Western-predominant standards of health care [28, 31, 61–63]. Therefore, medical interventions, although well-intended, might not be responding to these women’s needs; instead, they are likely to rely on health care professionals’ educational backgrounds; in-field experience; professional ethical standards [62]; and in the support of the institution where they work (e.g., explicit policies on how to proceed). In an era of global migration, understanding sociocultural clashes between health care professionals and migrants is crucial to address medical treatment undermined by both incomprehension [64, 65] and lack of agreement concerning the meaning of health [66] among participants. This seems to indicate that efforts towards reasonable accommodation of these women’s unattended health care needs and mainstream culture are needed. In this vein, policies that translate these women’s needs into concrete health care practices are crucial to the delivery of culturally safe care [26, 30].

Our study has limitations. First, we conducted a single interview with each consenting participant. However, these interviews were in-depth and were flexible enough to follow ideas participants brought up for discussion. Data collection and analysis were therefore built around emerging ideas and not merely attached to previous evidence. While it can be argued that only participants who have an interest in migrant populations might have consented to participate, it should be noted that of the total potential participants invited to participate (*n* = 68), only five health care professionals contacted did not participate (participation rate = 92.6%). In addition, by qualitatively interviewing health care professionals, the present study unveiled the voices of those who are actually in charge of medical practices, a side little explored until now. To better portray these voices, we presented a detailed account of themes discussed, and more importantly, a first-person vignette portraying common patterns we identified in health care professionals’ accounts that showed how personal and social factors identified in previous epidemiologic studies are intertwined and contribute to the creation of a complex process. This evidence complements previous works in which language was found to be a crucial obstacle regarding migrant women’s health care-related experiences [61, 64, 67–71]. Future research in this area is promising because it aims at unveiling how professionals’ perceptions are translated into their practices [72].

Findings from our study offer a springboard for future research. First, while the consideration of factors related to different levels of responsibility is important, it is also crucial to unveil how these factors interact [23]. Longitudinal research designs may facilitate both an understanding of how the process of acculturation by newly-arrived migrant women influences: (a) their use of maternity services [56] and (b) health care professionals responses to these women’s needs. In addition, research synthesis offer a clearer sense of newly-arrived migrant women’s journeys when looking for maternity care. This information may contribute to the design of inter-cultural interventions tailored for health care professionals, including, for example, specific basic university education and, once hired, continuing education; the creation of new policies and procedures and a space to discuss how available policies translate into interventions; and tailored tools to appropriately intervene. It would also be interesting to explore what barriers and facilitators health care professionals experience when working with migrant women and explore how these affect their practice. In addition, in this study we solely interviewed health care professionals, so interviewing migrant women as well as other health care professionals such as midwives would help illuminating and understanding the phenomenon we described. Notable, midwives were not included in this sample because only few midwives attend births in hospitals; moreover, in Quebec, midwives are not employees of the hospitals and are mainly work in birthing centres.

## Conclusion

According to health care professionals, newly-arrived migrant women face several challenges that go beyond maternity care and are difficult to address because they are deeply entangled with values and practices intrinsic to the health care system. For this reason, health care professionals might not be fully aware of all the challenges these women face and the health care system perpetuates [73]. Our study unveils that more efforts are needed to sensitise health care professionals to be more responsive to the maternity care needs and concerns of migrant women. Although the underlying reason may be that colonial values ruling marginalisation are largely entrenched in the health care system [58]; as for today, it seems that as portrayed by Molière in the *The Doctor in Spite of Himself* more than 400 years ago, health care professionals have a power associated with their status that they can use to either ‘dictate health’ or provide culturally safe health care services. The question that still remains unanswered is how aware are health care professionals of the power they have in their hands.
